# Takotsubo syndrome induced by severe hyponatraemia in mineralocorticoid-responsive hyponatraemia of the elderly: a case report

**DOI:** 10.1093/ehjcr/ytae513

**Published:** 2024-09-14

**Authors:** Fuyuki Asano, Daisuke Wakatsuki, Ayumi Omura, Hiroshi Suzuki

**Affiliations:** Division of Cardiology, Fujiyoshida Municipal Hospital, 7-11-1 Kamiyoshida-Higashi, Fujiyosida, Yamanashi 403-0032, Japan; Division of Cardiology, Fujiyoshida Municipal Hospital, 7-11-1 Kamiyoshida-Higashi, Fujiyosida, Yamanashi 403-0032, Japan; Division of Cardiology, Department of Internal Medicine, Showa University Fujigaoka Hospital, 1-30 Fujigaoka, Aoba-ku, Yokohama, Kanagawa 227-8501, Japan; Division of Cardiology, Department of Internal Medicine, Showa University Fujigaoka Hospital, 1-30 Fujigaoka, Aoba-ku, Yokohama, Kanagawa 227-8501, Japan

**Keywords:** Takotsubo syndrome, Severe hyponatraemia, Mineralocorticoid-responsive hyponatraemia of the elderly, Acute cardiac dysfunction, Case report

## Abstract

**Background:**

There are limited reports on mineralocorticoid-responsive hyponatraemia of the elderly (MRHE), a condition that can cause severe hyponatraemia.

**Case summary:**

An 85-year-old woman presented with transient loss of consciousness and nausea likely due to untreated severe hyponatraemia (114 mEq/L). Thirty-nine hours after initial admission, she developed sudden cardiac dysfunction and entered a circulatory collapse state. The patient was diagnosed with Takotsubo syndrome. Her hyponatraemia was an essential feature of syndrome of inappropriate antidiuretic hormone secretion. However, she was clinically hypovolaemic. Therefore, the hyponatraemia was diagnosed as MRHE. The serum sodium level was corrected with 3% hypertonic saline administered at a rate of 10 mL per hour, with careful monitoring to avoid overly rapid correction and prevent osmotic demyelination. After 14 days, her serum sodium level, electrocardiogram findings, and cardiac contractions on echocardiography improved.

**Discussion:**

To our knowledge, this is the first documented case of Takotsubo syndrome induced by severe hyponatraemia resulting from MRHE. The present report shows that acute cardiomyopathy can develop when severe hyponatraemia is not treated within several hours and at least a day. Since patients with MRHE are hypovolaemia statement, avoidance of diuretic drugs and water restriction for the treatment of hyponatraemia should be carefully considered, especially if they have acute cardiac dysfunction. This report highlights the need for prompt management of severe hyponatraemia in elderly patients and calls for further research on MRHE treatment protocols and its link to cardiomyopathy.

**Learning points**

- Takotsubo syndrome can be caused when treatment for severe hyponatraemia with a loss of consciousness is delayed.
- Mineralocorticoid-responsive hyponatraemia in the elderly is a rare hyponatraemia disease, and it is important to evaluate intravascular volume for a differential diagnosis.
- Diuretic therapy and water restriction should be considered carefully in patients with mineralocorticoid-responsive hyponatraemia of the elderly, especially in the presence of acute cardiac dysfunction.

## Introduction

Hyponatraemia, defined as a serum sodium concentration < 135 mEq/L, is the most common electrolyte imbalance encountered in clinical practice. Severe hyponatraemia presents with vomiting, cardiorespiratory distress, abnormal and deep somnolence, seizures, and coma due to cerebral oedema and increased intracranial pressure.^1^ Syndrome of inappropriate antidiuretic hormone secretion (SIADH) is a leading cause of severe hyponatraemia in adults.^2^ On the other hand, mineralocorticoid-responsive hyponatraemia of the elderly (MRHE) causes hyponatraemia and was first reported as a disease requiring differentiation from SIADH among older adults with hyponatraemia^3^; the case met the diagnostic criteria for SIADH but exhibited clinical hypovolaemia. Reports regarding patients with MRHE are limited.^4^

Takotsubo syndrome develops due to mental and physical stress and generally has a good prognosis.^5,6^ In rare cases, Takotsubo syndrome develops due to physical stress caused by hyponatraemia.^7–9^ This case report illustrates a rare but serious complication of MRHE-induced hyponatraemia, resulting in Takotsubo syndrome, and emphasizes the need for awareness of such potential outcomes in elderly patients.

## Summary figure

**Table:** 

−5 days	Onset of a transient loss of consciousness and nausea
−43 h	Hospitalization to previous hospital and confirmation of severe hyponatraemia (114 mEq/L)
−4 h	Appearance of chest pain. Electrocardiogram changed to exhibit negative T waves in limb leads I, II, III, and aVF, and the chest leads
0	Transfer to our hospital Confirmation of circulation collapse and severe hyponatraemia remains (117 mEq/L) Diagnosis with mineralocorticoid-responsive hyponatraemia of the elderly as the cause of hyponatraemia Diagnosis with Takotsubo syndrome as the cause of cardiac dysfunction
+7 h	Commenced on treatment of hyponatraemia including with 3% saline infusion
+14 days	Improvement of sodium level to normal range Improvement of the abnormal wall motion on echocardiography
+30 days	Fully recovered on electrocardiogram findings
+2 years	No relapse of the hyponatraemia and Takotsubo syndrome

## Case presentation

**Figure 1:**
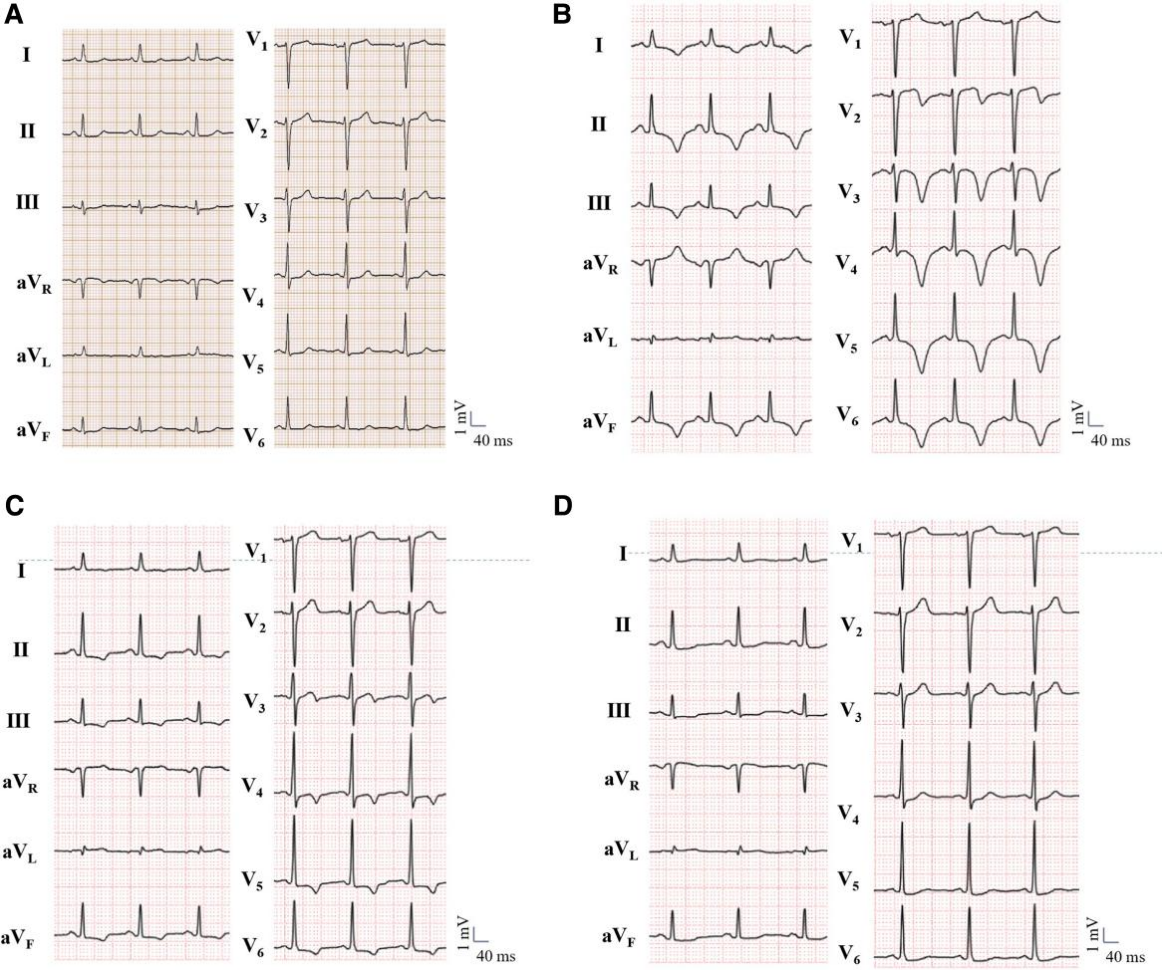
(*A*) ECG findings during the previous admission. (*B*) ECG findings on admission to our hospital. (*C*) ECG findings 14 days after admission. (*D*) ECG findings 30 days after admission. ECG, electrocardiogram.

The patient was an 85-year-old woman undergoing treatment for diabetes mellitus with sitagliptin phosphate hydrate (50 mg/day) and metformin hydrochloride (500 mg/day). She experienced repeated episodes of transient loss of consciousness and nausea 3 days prior and was admitted to a different hospital. A blood test on admission revealed a low serum sodium concentration of 114 mEq/L; however, the hyponatraemia was not treated. Electrocardiography results were within the normal range (*Figure 1A*). Thirty-nine hours post-admission, she experienced chest pain, and her electrocardiogram showed QT prolongation (QTc = 536 ms) and negative T waves in the limb leads I, II, and III, aVF, and chest leads (*Figure 1B*). Blood tests revealed elevated cardiac enzyme levels. She was transferred to our hospital because of suspected acute coronary syndrome. At the time of admission to our hospital, her height was 150 cm, weight was 40.7 kg, blood pressure was 95/69 mmHg, pulse rate was 95/min, SpO2 was 99% (room air), and body temperature was 35.7°C. Although the patient was fully conscious, her physical examination revealed signs of hypovolaemia, including a dry tongue, cold extremities, and orthostatic hypotension, as evidenced by the loss of consciousness upon sitting. An emergency coronary angiography revealed no significant coronary artery stenosis. Brain computed tomography revealed no bleeding or traumatic changes. Echocardiography revealed hypercontraction at the base of the heart and decreased contraction at the apex (see Supplementary material online, Echo clips  *S1A* and *S1B*). Therefore, the patient was diagnosed with Takotsubo syndrome. Blood test results at admission are shown in *Table 1*. The serum sodium level was markedly low (117 mEq/L). Urine osmolality was >100 mOsm/kg, urine sodium concentration was >30 mmol/L, and effective serum osmolality was <275 mOsm/kg; adrenal, thyroid, pituitary, or renal insufficiency was not observed. She was on a diet with normal salt and water intake and had not recently used diuretic agents. Decreased fractional sodium and urea excretions were observed. Although her haematocrit was within the normal range, iron studies revealed iron deficiency without anaemia, which may have contributed to the subtle clinical presentation of hypovolaemia. Echocardiographic findings on admission are shown in *Table 2*. Echocardiography revealed signs of hypovolaemia, including low ratio of the early diastolic transmitral flow to the late diastolic transmitral flow (E/A ratio), deceleration time and deceleration time, and ratio of the early diastolic transmitral flow to the early diastolic tissue Doppler mitral annular velocity (E/e′ ratio), a small inferior vena cava (IVC) dimension and a significant change in IVC diameter with a sniff (>50%). These echocardiographic indicators, combined with the clinical signs of hypovolaemia, supported the diagnosis of MRHE over SIADH. On the day of the hospitalization, she received treatment with a 3% saline infusion at a rate of 10 mL per hour for hyponatraemia. The serum Na level, signs of heart failure, and state of consciousness were checked after the first 4 h. The symptoms improved and the serum Na level increased by 5 mmol within 2 days, and on Day 3, it had increased by 10 mmol from the initial admission. Dietary intake (salt 6 g/day) was started on Day 2 of the hospitalization, and after 3 days, the dietary salt intake was changed to 7–8 g/day, and the saline infusion was terminated. The gradual improvement in sodium levels to 136 mEq/L by Day 14 reflects the controlled correction achieved through careful administration of 3% saline, preventing overly rapid shifts in serum sodium concentration. And, the negative T waves of the chest lead improved on electrocardiography (*Figure 1C*) and the abnormal wall motion were improved on echocardiography (see Supplementary material online, Echo clips  *S2A* and *S2B*). After 30 days, electrocardiography showed full recovery without cardiac medication administration (*Figure 1D*), indicating a transient decrease in left ventricular function. She visited the clinic for a check-up, and her blood tests were monitored every month for three months after discharge from our hospital. Thereafter, she visited the clinic to be checked for her condition every two months and underwent blood testing every five months. After 2 years, no brain damage, relapse of hyponatraemia, or Takotsubo syndrome occurred.

**Table 1:** Laboratory values on admission

	Results	Reference range	
WBC	10 040	33–90	/μL
Hgb	13.3	11.5–15.5	g/dL
Hct	39.1	34.5–46.0	%
BUN	17.5	8–20	mg/dL
Cre	0.99	0.46–0.79	mg/dL
UA	4.9	2.6–5.5	mg/dL
Na	117	138–145	mEq/L
K	5.2	3.6–4.8	mEq/L
OSM	245	275–290	mOsm
BNP	191.1	<18.4	pg/mL
Troponin T	0.298	0–0.1	ng/mL
Plasma antidiuretic hormone	2.1	<4.0	pg/mL
Adrenocorticotropic hormone	8.6	7.2–63.3	pg/mL
Plasma cortisol	32.9	3.7–19.4	μg/dL
Plasma renin activity	12.5	0.2–2.3	ng/mL/h
Plasma aldosterone	180	36–240	pg/mL
Thyroids stimulating hormone	1.88	0.35–4.94	μU/mL
Free T3	2.19	1.71–3.71	pg/mL
Free T4	1.46	0.70–1.48	ng/dL
Urine osmolality	554	50–1300	mOsm
Urine sodium	39		mEq/L
Fractional sodium excretion	0.4		%
Fraction urea excretion	2.7		%
Fraction uric acid excretion	3.7		%

WBC, white blood cell; Hgb, haemoglobin; HcT, haematocrit; PLT, platelet; CRP, C-reactive protein; BUN, blood urea nitrogen; Cre, serum creatinine; Na, serum sodium; CL, serum chloride; K, serum potassium; OSM, serum osmolality; BNP, brain natriuretic peptide.

**Table 2:** Echocardiography parameters

On the day of hospitalization		
EF	47	%
LVDD	35	mm
LVDS	20	mm
LVEDV/BSA	36	mL/m^2^
LVESV/BSA	19	mL/m^2^
SV/BSA	16	mL/m^2^
LAV/BSA	18	mL/m^2^
E velocity	36	cm/s
A velocity	82	cm/s
E/A ratio	0.44	
DcT	305	ms
E/e′ ratio (septum)	10	
TRPG	16	mmHg
Dimension of IVC	8.3	mm
Change in the diameter of the IVC with a sniff	>50	%

EF, ejection fraction; LVDD, left ventricular diastolic diameter; LVDS, left ventricular systolic diameter; LVEDV, left ventricular end-diastolic volume; BSA, body surface area; LVESV, left ventricular end-systolic volume; SV, stroke volume; LAV, left atrial volume; E/A ratio, ratio of early diastolic transmitral flow (E-wave) to late diastolic transmitral flow (A wave); DcT, deceleration time; E/e′ ratio, the ratio of early diastolic trans mitral flow (E-wave) to the early diastolic tissue Doppler mitral annular velocity (e′); TRPG, tricuspid pressure gradient; IVC, inferior vena cava.

## Discussion

Herein, we present a case that developed a circulatory collapse in Takotsubo syndrome due to MRHE-induced severe hyponatraemia. Severe hyponatraemia is defined as a serum sodium concentration < 125 mmol/L.^1^ The patient was determined to have severe hyponatraemia in admission on previous hospital. Many causes of severe hyponatraemia in adults include thiazide use, post-operative state, other causes of SIADH, polydipsia in psychiatric patients, and transurethral prostatectomy.^10^ In contrast, three cases of MRHE indicating it as a disease requiring differentiation from SIADH were reported.^3^ The diagnosis of MRHE in the previous report was based on ‘hypovolemic hyponatremia with fulfilled criteria for SIADH’.^4^ The patient’s right atrial pressure was estimated to be 0–5 mmHg on echocardiography because the dimensions of the IVC were small and a change in the IVC with a sniff was <50%.^11^ Moreover, the left ventricular filling pressure was low, and the left ventricular volume was not increased.^12,13^ Hypovolaemia was assessed by physical examination (hypotension and loss of consciousness with head elevation), suppression of fractional sodium, urea excretion, and echocardiography. The haematocrit value would have been higher if the patient was dehydrated. However, we considered that it was not high because of iron deficiency without anaemia.^14^ In patients with severe cardiac failure or SIADH, water restriction or diuretic drug infusion should be considered.^1,10^ However, these were avoided in the present case because of hypovolaemia. Therefore, differentiating MRHE and SIADH is important. Although the patient met the SIADH criteria, the clinical and echocardiographic evidence of hypovolaemia led to the diagnosis of MRHE-induced hyponatraemia. This distinction was crucial as it directly impacted the treatment approach, avoiding the use of water restriction or diuretics, which could have exacerbated her condition.

We diagnosed Takotsubo syndrome using an InterTAK Diagnosis score of 56 points (female sex, physical stress, and electrocardiography findings)^15^ and confirmed transient decreased left ventricular function.^16^ Several reports have described cases of severe hyponatraemia-induced Takotsubo syndrome.^7–9^ In one case, the authors mentioned that the severity of the hyponatraemia seemed to be important for the occurrence of Takotsubo syndrome.^9^ Interestingly, present patient developed Takotsubo syndrome 39 h after the initial presentation, despite a slight improvement in serum sodium levels from 114 to 117 mEq/L. This suggests that persistent stress from the underlying hyponatraemia, rather than the severity alone, may have contributed to the cardiac event. In patients with strokes, the sympathetic nervous system can directly release catecholamines and thereby induce cardiomyocyte toxicity. A long-term elevation of serum catecholamines produces cardiotoxicity and cardiac dysfunction.^17^ In the present case, we speculated that prolonged hyponatraemia-induced cerebral oedema likely triggered a sustained release of catecholamines, leading to cardiotoxicity and subsequent Takotsubo syndrome.

To our knowledge, this is the first report of clinically diagnosed Takotsubo syndrome caused by MRHE-induced severe hyponatraemia. Previously, chronic symptomatic hyponatraemia in older postmenopausal women was associated with major morbidity and mortality, and intravenous sodium chloride was associated with favourable outcomes.^18^ On the other hand, the most frequent complication of Takotsubo syndrome is heart failure.^19^ Throughout the saline infusion, the patient was closely monitored for signs of fluid overload and neurological symptoms, ensuring a safe and effective correction of the hyponatraemia. This case also illustrates the delicate balance required in managing MRHE with concurrent Takotsubo syndrome, where aggressive sodium correction must be weighed against the risk of heart failure.

In conclusion, we report a rare case of Takotsubo syndrome caused by MRHE-induced severe hyponatraemia. This case highlights the need for prompt and appropriate correction of sodium levels while carefully managing the patient’s fluid status to prevent fatal outcomes. This case underscores the importance of differentiating MRHE from SIADH in elderly patients with hyponatraemia, as well as the need for vigilant monitoring and timely intervention to prevent severe complications such as Takotsubo syndrome.

## Lead author biography


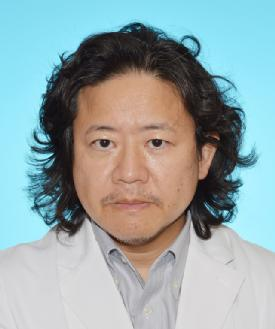


Fuyuki Asano is a Japanese medical doctor and cardiologist from the Fujiyoshida Municipal Hospital. He has an interest in cardiology and catheter intervention.

## Supplementary Material

ytae513_Supplementary_Data

## Data Availability

The data that support the findings of this study are available from the authors upon reasonable request.

## Supplementary material

**Consent:** The authors confirm that written consent for the submission and publication of this case report, including the images and associated text, has been obtained from the patient, in line with COPE guidance.

**Funding:** None declared.
